# The Last Ten Years of Advancements in Plant-Derived Recombinant Vaccines against Hepatitis B

**DOI:** 10.3390/ijms17101715

**Published:** 2016-10-13

**Authors:** Young Hee Joung, Se Hee Park, Ki-Beom Moon, Jae-Heung Jeon, Hye-Sun Cho, Hyun-Soon Kim

**Affiliations:** 1 School of Biological Sciences & Technology, Chonnam National University, Gwangju 61186, Korea; yhjoung@chonnam.ac.kr (Y.H.J.); shpark860@daum.net (S.H.P.); 2 Molecular Biofarming Research Center, Korea Research Institute of Bioscience & Biotechnology (KRIBB), Daejeon 34141, Korea; irony83@kribb.re.kr (K.-B.M.); jeonjh@kribb.re.kr (J.-H.J.); hscho@kribb.re.kr (H.-S.C.)

**Keywords:** antigen, hepatitis B virus, plant edible vaccine, recombinant protein, molecular farming, virus-like particles

## Abstract

Disease prevention through vaccination is considered to be the greatest contribution to public health over the past century. Every year more than 100 million children are vaccinated with the standard World Health Organization (WHO)-recommended vaccines including hepatitis B (HepB). HepB is the most serious type of liver infection caused by the hepatitis B virus (HBV), however, it can be prevented by currently available recombinant vaccine, which has an excellent record of safety and effectiveness. To date, recombinant vaccines are produced in many systems of bacteria, yeast, insect, and mammalian and plant cells. Among these platforms, the use of plant cells has received considerable attention in terms of intrinsic safety, scalability, and appropriate modification of target proteins. Research groups worldwide have attempted to develop more efficacious plant-derived vaccines for over 30 diseases, most frequently HepB and influenza. More inspiring, approximately 12 plant-made antigens have already been tested in clinical trials, with successful outcomes. In this study, the latest information from the last 10 years on plant-derived antigens, especially hepatitis B surface antigen, approaches are reviewed and breakthroughs regarding the weak points are also discussed.

## 1. Introduction

Hepatitis B (HepB) is an infection with the hepatitis B virus (HBV), which attacks the liver and can cause both acute and chronic disease. The World Health Organization (WHO) estimates that 240 million persons are chronically infected with HBV and that more than 780,000 people die every year due to complications of HepB, including cirrhosis and liver cancer [1]. The point that needs the most attention is the high rates of chronic infection found in the sub-Saharan Africa and East Asia, where between 5% and 10% of the adult population is infected, as well as in the Amazon and the southern parts of eastern and central Europe. Otherwise, less than 1% of the population in Western Europe and North America is chronically infected [1].

HBV is a hepatotropic DNA virus that replicates by reverse transcription. The human HBV is a small circular DNA molecule of 3.2 kb [2]. Its genome consists of four partially overlapping open reading frames (ORFs), namely the envelope gene (*pre*-*surface*/*surface* (*pre*-*S*/*S*)), the core gene (*pre*-*core*/*core* (*pre*-*C*/*C*)), the polymerase gene (*pol*) and the transactivating protein X (*X*). The ORF *pre*-*S*/*S* encodes pre-S1, pre-S2 and surface (S) proteins that form the surface antigen (HBsAg), and HBsAg protein is the main antigen to elicit virus-neutralizing and protective antibodies by the immune system [3,4]. Understanding the HBV genome and structure is an essential prerequisite for preventive or therapeutic vaccination against HepB.

## 2. Approach for HBV Vaccine

A vaccine against HepB has been available since 1982. This first licensed anti-HBV vaccine containing subviral particles of HBV purified from the inactivated serum of carriers revealed very high efficacy [5], and a subsequent subunit vaccine made using the small surface antigen (S-HBsAg) was developed in the early 1980s [6]. The yeast system for the recombinant antigen was to ensure safety and low cost. Yeast-derived S-HBsAg assembled into virus-like particles (VLPs) were as immunogenic as natural subviral particles, and highly effective vaccines containing S-HBsAg have been widely used as prophylactic vaccines against HBV infection [7]. However, some groups of vaccines do not develop protective immunity against the virus and immunosenescence frequently occurs in adults [8]. Additionally, high cost limit and the necessity of accompanying infrastructure for the cold chain distribution and intravenous administration still constituted a barrier to vaccination approaches in developing countries. In order to successfully solve these problems, many research projects have been undertaken to develop more efficacious, easily administrated, and thermostable vaccines.

A new recombinant HBV vaccine containing the *pre*-*S*/*S* has greater immunogenic potential than the conventional S antigen-based vaccines in terms of antibody induction and cellular immune response. Middle (pre-S2 + S, M-HBsAg) or large (pre-S1 + pre-S2 + S, L-HBsAg) surface antigens [9] have been used as components of specific immunotherapeutic vaccines for chronic HBV carriers [10,11]. Additionally, chimeric protein created by fusing the HBV core antigen (HBcAg) to the pre-S1 showed strong anti-HBc and moderate anti-pre-S1 immune responses [12].

## 3. Plant-Based Expression System for Vaccine Development

Although vaccination is one of the most powerful and cost-competitive achievements, some vaccines may still have certain limitations related to maintenance of the cold chain, downstream processing costs, administration risk, and expensive scalability [13,14,15,16,17]. From these reasons, the use of plant cells as alternative production platforms have received considerable attention in terms of intrinsic safety, scalability, and posttranslational modification of target proteins [17,18]. Plant systems can be scaled up quickly to generate large quantities of the protein product, are not susceptible to contamination with known human or mammalian pathogens and are resistant to enzymatic digestion in the gastrointestinal tract. In addition, transgenic plants can be engineered to express and translate multiple proteins concurrently with appropriate folding and assembly into multimeric proteins, especially the posttranslational adjustments of antibodies. Not all recombinant antigens will benefit from plant-based systems, but the best production system for each recombinant protein should be chosen using a case-by-case approach [19]. Merlin et al. [19] proposed that plants are the most the beneficial for the production of four major categories of pharmaceutical proteins: ones that are required in large quantities, that need to be rapid-response, that require complex posttranslational modifications, or that are intended for oral delivery. Within these categories, they suggested appropriate candidates to meet a spectrum of research, development, commercial needs, such as human glutamic acid decarboxylase, Norwalk virus-like particles, monoclonal antibody 2G12, and human interleukin-6.

Those plant-made antigens have already been tested in clinical trials, with successful outcomes (Table 1). The enzyme glucocerebrosidase for Gaucher’s disease, the first PMF-derived enzyme “ELELYSO™”, has been approved and marketed by Protalix in 2012. ELELYSO™ is based on the use of carrot cells to produce recombinant taliglucerase alpha, which is used in enzyme replacement therapy to treat adult patients. This special Food and Drug Administration (FDA) approval case was fast tracked based on its applicability to a rare genetic disease and the bioreactor production under stringent conditions [20,21]. Medicago has ongoing phase II clinical trials for a plant-derived VLP quadrivalent seasonal influenza vaccine and an H5 pandemic influenza vaccine [22]. They are focusing on VLP vaccine development. VLPs are self-assembled structures derived from viral antigens that mimic the native architecture of viruses but lack the viral genome and thus are not infective. Another important advantage as emerging vaccine is the more effective activation of key aspects of the immune response to achieve potent immune stimulation and to provide immunological memory for long-lasting protection [22,23]. Several reports on the expression of properly assembled VLPs at adequate levels have been published for the influenza virus [24], human papillomavirus [25], human immunodeficiency virus [26,27], Norwalk virus [28], and hepatitis B virus [26,29,30]. In recent, VLPs have been applied in various fields, not only as vaccines [31,32] but also as delivery agents for therapeutic medicine [33,34,35,36]. In addition, Paul et al. [37] summarized four major developments of molecular pharmaceuticals occurring in the world. More recently, Yao et al. [21] reviewed the challenges and opportunities of plant molecular farming, including various examples of clinical trials from the perspectives of biosafety, appropriate expression systems, and possible and potential application.

Plant-based platforms including whole plant, organs or cell and expression technology to produce target antigens of interest are diverse [38,39,40]. Representative plant species expressing the oral vaccine are potato, tomato, and tobacco; additionally, maize, rice, carrot, and soybean are also applied in this field [41,42,43,44,45,46,47,48,49]. Those plants are mainly focused on traditional and usually eaten crops in human, because it is known that inexperienced plants sometimes have problems with certain plant allergies. Target antigen proteins were expressed by a plant cell nuclear genome expression system in these plant species. Edible plant vaccines are based on different parts of plants, such as fruits, seeds, and root vegetables. Such food vaccines are prepared directly without expensive purification of the antigens, which is essentially required for parenteral administration of vaccines [50]. Therefore, the lyophilization of organs expressing stable antigens would facilitate their processing, purification and storage, reducing costs and allowing more practical vaccines. Although stable transformation into transgenic plants is commonly accepted, the low production level of the resultant recombinant protein remains an issue of concern. An efficient alternative to nuclear transformation for vaccine antigens and other therapeutic proteins is plastid transformation [51]. The highest expression of transgenes, up to more than 70% of total soluble protein, are reported in chloroplast transformation [52,53] Otherwise, the universal expression level in most studies has been 1% of total soluble protein (TSP) or 50 µg/g fresh leaf tissue [54,55]. Chloroplast technology can also avoid the controversy related to transgene containment [40] and express multigenes as single operon [56]. Waheed et al. [40] reviewed recent vaccine antigens against human diseases expressed via plastid genome since 2011. Two plant species, tobacco (15 different antigens) and lettuce (four different antigens), have mainly been used in plastid transformation. These results suggest that more industrial interest is needed to strengthen the research/academia-industry linkages in the chloroplast-based vaccine market.

Stable transformation has its own advantages such as reliable harvest of target proteins over multiple generations and optimized protocols for delivery of foreign genes into various plant species. Although there are problems with the time required, seed resources can be grown anywhere with minimal cost and labor once the plant has been developed for the first time [21].

Most clinical trials, except for the three cases of ELEYSO, PRX-102, and recombinant human intrinsic factor, have used a tobacco-based transient expression system (Table 1). In recent years, interest in transient expression has increased due to the containment of the system and the possibility of rapid upscaling due to the short interval between transformation and expression, which are attractive features for the industrial scale production and approval of the expressed products, e.g., the mass production of tobacco by Medicago and Kentucky BioProcessing. Pogue et al. [57] reviewed plant-based transient expression systems for the production of pharmaceutical-grade recombinant aprotinin and a monoclonal antibody product. Transient expression provides a safe and environmentally friendly system for both indoor and outdoor application with high speed and low cost of the genetic manipulation, rapid manufacturing cycles, and economical production. Transient production using an *Agrobacterium tumefaciens-*mediated transfer-DNA delivery system (Agro-infiltration) and/or virus-based replicating systems, the two dominant approaches, guarantees both the quality of the resulting purified products and the speed of development [57,58]. Spiegel et al. [59] demonstrate the application of the classical *Nicotiana benthamiana/A. tumefaciens* transient expression system to accelerate the development of a malaria vaccine candidate, with screens for expression, solubility, and stability using fluorescent fusion proteins. In Marin Viegas et al. [60], a transient expression system for the production of human TG2 in *N. benthamiana* leaves was optimized, and the reactivity of plant-produced TG2 in a CD screening test was evaluated. Hence, transient expression performed in contained facilities satisfies good manufacturing practices, and quick expression can avoid the time-consuming stable transformation [58,61]. In a comparison of productivity in terms of biomass production, Hiatt and Pauly [62] reported that grams of product may take only two weeks plus a few weeks more. In large-scale biomanufacturing systems, recombinant proteins can be produced at levels of 200–1000 mg/kg fresh weight tissue in as little as three months [57]. The transient expression of human Interleukin-6 in *N. benthamiana* (7.8% TSP) produces 80-fold more than stable expression in tobacco plants (0.3 mg/g fresh weight (FW)) [63]. Conversely, hGAD65mut is expressed at higher levels in stable tobacco plants (143.6 µg/g FW) than in *N. benthamiana* (96.6 µg/g FW) [64]. This result suggests that expression potential or level varies case by case, depending on the target protein.

## 4. Studies on Plant Vaccines

In last decade, there has been a considerable increase in the use of transgenic plants to generate recombinant proteins for medical and veterinary use (Table 2). Research groups worldwide have attempted to develop more efficacious plant-derived vaccines for over 30 diseases, most frequently hepatitis B and influenza. In the case of hepatitis B, both stable and transient expression systems have been developed in various plants, including potato, lettuce, tobacco, tomato, carrot, and Arabidopsis for stable expression and *N. benthamiana* for transient expression. A detailed review of hepatitis B will be presented in the next part of this article.

Influenza is also a main target of this field because it is a widely distributed viral infection of humans and animals, and a new epidemic strain appears every one to two years. This pattern requires the production of new vaccines at the same frequency, and a promising solution is to establish a rapid, flexible, and safe system. The production of various antigens such as hemagglutinin (HA) and the extracellular domain of matrix protein 2 (M2e) has mainly focused on transient expression systems using *N. benthamiana* leaves. Using the Medicago “Proficia™” system, vaccine production can be initiated within less than three weeks from the identification of the genetic sequence of a pandemic or seasonal influenza strain [155].

Vamvaka et al. [74] reported the development of transgenic rice plant expressing the HIV-neutralizing antibody 2G12 in the endosperm (42 µg/g dry seed weight) to evaluate the potential of rice seeds as a vehicle for inexpensive microbicide production. Production is higher than the initial achievement of maize-derived 2G12 (30 µg/g) by Rademacher et al. [156]. Rubio-Infante et al. [75] demonstrated the immunogenic potential of tobacco chloroplast-derived multi-HIV in an oral immunization scheme and proposed it as a vaccine prototype capable of inducing broad immune responses as it carries various B and T cell epitopes from several HIV strains.

Dengue has become a significant public health problem, and the threat of Dengue fever is now increasing in temperate regions due to dramatic climate change. The rice codon-optimized consensus domain III of dengue virus envelope glycoprotein (E) has been fused to the M cell-binding peptide via agroinfiltration with a plant virus-based expression system [157,158,159]. Carrying these results a step further, Kim et al. [159] generated an Ebola RIC-based DENV vaccine in tobacco plants using a geminiviral vector expression system and reported its immunogenic properties as a self-adjuvanting dengue vaccine candidate. Previously, Phoolcharoen et al. [160] reported that plant-expressed Ebola RIC protected mice against a lethal Ebola virus challenge.

The expression of subunit vaccines for animal viral diseases, such as avian influenza [98,161], foot-and-mouth disease (FMD) [100,101,102], and diarrhea [123,126,127], which are considered to be the most important causes of economic losses in plants, has been frequently reported. The commercialization of veterinary vaccine is relatively easy compared with that of human vaccines. To date, four cases in clinical trials are ready to enter the market. Recombinant plant viral hamagglutinin-neuraminidase (HN) protein of the Newcastle disease virus (approved by United States Department of Agriculture (USDA)) and a mixture of antiviral vaccines have been prepared by Dow Agro Sciences. A plant anti-rabies vaccine (completion of phase I) has been developed by Thomas Jefferson University. Chicken coccidiosis is in the second phase of clinical trials run by the Canadian Guardian Biosciences company. For the FMD vaccine, *N. benthamiana*, tomato, and rice have been used for both transient and stable transformation. Most recently, Muthamilselvan et al. [99] have presented an important advance with 2.1 mg/20 g fresh weight of plant callus-based biomass in the cost-effective large-scale production of immunopeptide vaccines against FMD. They established transgenic cell-suspension cultures from *N. benthamiana* leaves expressing FMDV VP1 and gene silencing suppressor p19 and then validated the efficacy using immunized guinea pigs that produced humoral antibodies. Lim et al. [100] developed an SYCMV-derived vector containing FMDV VP1, which was expressed successfully in soybean plants by infiltration.

Diarrhea, a representative infectious disease caused by rotavirus and diarrhea virus, is also being studied. Various groups have expressed rotavirus capsid proteins (VP2, -4, -6, -7, and -8) in plants. Most studies have introduced VP6, which forms the intermediate capsid, as a vaccine epitope into different plant species, including *N. benthamiana*, tomato, and *Medicago sativa*. Pera et al. [162] suggest that the highly immunogenic VP8 epitopes produced in *N. benthamiana* are candidates for a subunit vaccine, specifically for the G9P rotavirus strain.

## 5. Production of HBV Antigens in a Plant System

The greatest problem of plant-derived vaccine development is the extremely low expression level of the foreign protein in plants. For this reason, many researchers have studied how to improve protein expression levels in plants. In the case of plant-derived HBV vaccines, the first report was on the expression of the small hepatitis B surface antigen (S-HBsAg) in transgenic tobacco plants. In this report, the HBsAg produced in transgenic tobacco was antigenically and physically similar to the HBsAg particles derived from human serum and recombinant yeast [163]. Afterward, many research groups attempted HBsAg expression in different tissues and plant species, such as tobacco, potato, lettuce, soybean, lupine, maize, tomato, peanut and *Laminaria japonica* (Table 3). In the transgenic tobacco plant transformed with the S-HBsAg gene controlled by the 35S promoter, expression levels were very low: less than 0.01% total soluble protein and less than 10 ng/g fresh weight in leaf tissues. The expression levels of S-HBsAg in other plant species were not significantly higher; in some species, expression levels were even lower than in tobacco. To improve vaccine production in plants, the most widely used strategies involve: (1) suitable promoters, such as strong constitutive promoters, tissue-specific promoters and promoters that are inducible by environmental factors; (2) targeting systems to specific organelles; (3) optimized codon usage; (4) alternative polyadenylation signals; (5) increased translation efficiency using leader sequences; and (6) different vector systems. Many HBsAg-overexpressing transgenic plants have been developed using strong constitutive promoters, such as the 35S promoter with enhancer [164,165,166]. In addition to tissue-specific promoters, the patatin promoter for potato tuber [165,167], globulin promoter for maize seed [168] and fruit-specific promoters [169,170] were used. Specific organelle-, endoplasmic reticulum (ER)-, vacuole- and chloroplast-targeted strategies have also been tried [167,171].

The HBsAg has been expressed in non-edible plants, such as tobacco, using four different expression cassettes: the HBsAg gene without ER retention signal (HBS), the HBsAg gene with ER retention signal (HER), and each gene controlled by the ubiquitin promoter (UBQ) or ethylene forming enzyme promoter (EFE) [172]. In this report, the maximum expression level (19.4 ng/g FW of leaves) was observed in EFE-HBS transformed plant growth in vitro, but a higher proportion of the particulate form of the antigen was observed when it was expressed with an ER retention signal. The EFE promoter is more effective in in vitro-cultured plantlets, whereas the UBQ promoter is more effective in greenhouse-grown plantlets. The maximum expression level was 2 µg/g FW in the UBQ-HER transformed NT-l cell suspension culture [173]. The expression level was increased up to 8 µg/g FW using HBsAg fused with the 3′ region from the soybean vegetative storage protein gene and was controlled by a chimeric ocs-mas promoter. Upon transformation into a soybean cell culture using the same vector, the maximum expression level was 74 µg/g FW [174].

HBsAg has been expressed in vegetative crops, such as potato, tomato, soybean and lettuce. The expression level of transgenic potato tubers was 1–11 µg/g FW. The highest expression in a tuber was developed using a construct driven by the CaMV 35S promoter with dual enhancers, the tobacco etch virus 5′-UTR, and the 3′ region from the soybean vegetative storage protein gene [165]. Expression level of HBV-protein in potato was little increased when controlled by the tuber specific promoter [167]. Target DNA is inserted into a genomic DNA as a random event when a using *Agrobacterium*-mediated transformation. For this reason, it is difficult to conclude what is the best method for increase of HBV-protein expression level because differences in the expression level between the transgenic lines even with the same vector construction. Expression in tomato fruit has been reported at 0.5 µg/g dry weight. To achieve a higher level of expression, several strong and inducible promoters, such as the enhanced dual 35S, UBQ and EFE promoters, were tested, as well as organelle targeting sequences. The greatest improvement resulted from the HBsAg gene with an ER retention signal controlled by EFE promoter [169]. Sunil Kumar et al. [170] reported HBsAg transformation in banana. The maximum expression in banana leaves has been reported at 38 ng/g FW. The expression levels in banana fruits were not presented in this report, but the expression level was presumably lower than in the leaf tissue. As with leafy vegetables, a variety of expression technologies have not yet been applied. In lettuce, the maximum expression level was 60 µg/g FW, which is the maximum anti-HBsAg antibody titer of 300 mIU in immunized mice serum [188,189,201]. Upon transformation into a soybean cell culture using a construct of HBsAg fused with the 3′ region from the soybean vegetative storage protein gene and as controlled by a chimeric ocs-mas promoter, the maximum expression level was 74 µg/g FW [174].

Grains are a further option for the expression of candidate vaccine antigens. They have long stability of expressed recombinant proteins with low water content [212]. In maize seed, the maximum expression has been reported at 0.51% of total soluble protein (approximately 80 µg/g FW). This level of expression was achieved using a barley alpha amylase signal sequence-fused S-HBsAg gene with a 3× globulin1 promoter [168]. All of the results suggest that the expression levels of HBsAg are highly variable and depend on plant species, tissue types and culture conditions.

The major recombinant hepatitis B vaccines contain S-HBsAg; therefore, the expression of this protein has been the focus in plants. The proteins preS2-S, M-HBsAg, and preS1-preS2-S, L-HBsAg, have been much less studied than S-HBsAg. M-HBsAg and L-HBsAg have been transformed into potato, tomato, and tobacco (Table 3). Although the expression was optimized using suitable promoters, leader sequences and targeting signals, the expression levels of M-/L-HBsAg were lower than for S-HBsAg. However, HBcAg induces a heightened immune response [213,214] and spontaneously assembles into capsid-like particles [215]. For these reasons, efforts devoted to the production of an anti-HBV vaccine have focused on HBcAg in the last few years. Especially, HBcAg has been abundantly produced using transient expression systems mediated by ICON binary vectors [208] or viral vector systems [209] (Table 3).

## 6. Breakthroughs Regarding the Weak Points of Plant-Derived HBV Antigens as Injected Vaccines

Transgenic tobacco plants-derived HBsAg was antigenically and physically similar to the human serum and recombinant yeast derived-HBsAg particles [163]. To analyze the immunological response in vivo, tobacco-expressed HBsAg was purified and injected into BALB/c mice. The anti-HepB response to the tobacco-derived HBsAg was qualitatively similar to the response obtained by immunizing mice with commercialized yeast-derived HBsAg vaccine [175]. These results showed a possibility of developing injected vaccines using plant-expressed HBsAg.

Due to differences of the manufacturing processes between companies, the amount of HBsAg protein per dose differs among the various HBV vaccine products [216]. For this reason, there is no international standard for the HBsAg protein quantity in vaccines, but there is a standard based on protective efficacy of vaccination related to the anti-HepB antibodies induction. An anti-HBsAg of ≥10 mIU/mL measured 1–3 months after the last dose of the vaccine are considered to be immune to HepB. Although no international standard of antigen concentration is defined, considering the feasibility and cost-effectiveness of the injected vaccine, the concentration of antigen should be over 40 μg/mL [217].

Despite many attempts to increase HBsAg expression in transgenic plants, the expression level remains too low for use as an injected vaccine. The currently used HBV vaccine contains HBsAg and is produced by yeast cells. The yeast-derived HBV vaccine can be supplied inexpensively ($1–20 per single dose [218]); therefore, it is difficult for plant-derived vaccines to have a competitive price. However, plant suspension cultures may be used as an alternative to yeast to produce antigens for purification. Expression levels have approached 74 μg/g FW (22 mg/L culture medium) in transgenic soybean culture [174]. Although the expression level (8 μg/g FW) was lower in transgenic tobacco cell suspension culture than in soybean [174], the former has been used to secrete HBsAg into the culture medium, with a six-fold increase in secretion in response to jasmonic acid or salicylic acid treatment during cell culture, and the amount of antigen secreted was 180 μg/L medium [170].

Another breakthrough regarding expression problems was achieved through the utilization of virus-based transient expression systems for the robust production of HBV antigens, such as S-HBsAg and HBcAg, with yields as high as 2 mg/g FW [208,209,211]. Tobacco-derived proteins showing the maximum anti-HBsAg antibody titer of 1165 mIU in immunized mice serum [211] are preferred the application of injection after purification process, rather than oral administration in order to remove many toxic alkaloids and phenolic substances which have a tobacco plant [219]. However, improvements of several orders of magnitude are still needed for plant cell culture systems to be competitive, particularly given the slow growth rates of plant cells compared with yeast.

In transgenic suspension cell culture, the formation of VLPs by HBV antigens made it possible to exploit relatively inexpensive protein purification techniques, such as the sucrose gradient [174,182,184] or cesium chloride gradient ultracentrifugation [173]. The highest expressed soybean cell culture was used for antigen purification, and the antigen was suitable for injection [174], but the yield remained unsatisfactory and was not cost-effective.

## 7. Breakthroughs Regarding the Weak Points of Plant-Derived HBV Antigens as Oral Vaccines

The biggest advantage of edible plant-derived vaccines is their easy application to oral delivery. The benefits of plant-derived edible vaccines are as follows: (1) during oral delivery, plant-derived vaccines are protected in the stomach by plant cell wall and slow release in the gut; (2) the plant tissue expressing antigen may be used as raw or dried food; (3) capsules can also be made from partially or fully purified vaccine proteins; (4) no need for cold chain systems for storage and delivery of the plant tissues or extracts; and (5) the plant-derived vaccines are cost efficient compared with traditional vaccines.

Edible plant-derived HBV antigens have been administered by oral injection or feeding in mice with/without adjuvants [41,165,166,167,193]. An oral vaccine candidate has also been administered to human volunteers in small-scale clinical trials without adjuvants. The first trial was administered to three human volunteers in row lettuce leaves in two doses (0.5–1 μg of S-HBsAg/dose) without the use of an adjuvant. All volunteers responded, with two of them having serum responses in excess of the protective minimum level (10 mIU/mL of serum). However, the antibody levels declined rapidly [187,220]. In the second trial, previously vaccinated human volunteers were fed two or three doses of 100 g of raw potato tubers (approximately 1 mg of the S-HBsAg/dose). More than half of the subjects showed increased antibody titers [221]. The animal experiments and trials showed the potential for plant-derived HBV antigens to be used as an oral vaccine for the prevention of HBV, but there remain many problems to be solved for practical application, such as the administration of bulky plant material, declining long-term responses, individual differences in the immune response and the difficulty of defining the antigen dose [222].

The expression level of plant-derived HBV antigen is only 1/20–1/25 of the expression of yeast-derived HBV antigen; however, the expression yield and plant production scale are still increasing [223,224]. Tomato is possible intake without any processing or cooking. Therefore, tomato fruit is a very attractive crop to develop an oral vaccine. According to the study to date, the expression level of HBV antigen was very low as 10 ng/g FW (Table 3). The maximum titers of anti-HBsAg antibody in serum is 300 mIU using oral application. This antibody yield was high compared to the expression level of HBV antigen in tomato fruits [225]. HBV antigen expression in maize produced much higher levels of antigen, and the palatability and digestibility were better than for potato. That is, cereal crops can easily transport or storage in dry state. In addition, the maize system induced a strong immune response with 4632 mIU of maximum titer by both injection and oral administration [193,194]. This result suggests the possibility of providing a raw material for thermostable formulation at $0.01 per dose [193]. Plant components such as saponin, flavonoids, and plant oils also function as adjuvants [226,227,228] and help maintain the immune response in the long term [195].

The lyophilization method is an excellent way to increase the stability and shelf life of the plant-derived vaccines. In the previous study, the storage stability of lyophilized powder form was limited at 4 °C [189]. In a recent study, successful long-term storage at 37 °C was achieved though improvements in the process [229]. It is easier to control the concentration and standardize antigen doses and process the antigen into a tablet or capsule form using a powdered tissue instead of freeze-drying [188].

## 8. Conclusions

Despite over 20 years of effort, no commercial plant-based anti-HBV vaccine has been developed. To commercialize a plant-derived HBV vaccine, several points should be considered. First, the greatest barrier is the low expression levels of HBV antigen in plants; however, expression yield and plant production scale can still be increased using plant expression vector optimization, which should be focused on the target plant. The process can also be more competitive by improving the plant-derived antigen to increase the immune response to the vaccine. Second, an HBV antigen expressed in an edible plant has the advantage of being usable as an oral vaccine without processing. It is first necessary to analyze the characteristics of the target plants and the expressed protein for the development of an oral vaccine because plant components, secondary metabolites and foreign protein expression characteristics vary with plant species. To obtain feasible and cost-effective vaccines, the target plants for edible vaccines should have a long shelf life, be heat stable and be edible as a raw material. Candidate grain crops are maize and rice; candidate vegetative crops are tomato and banana. Third, consideration should be made of the public’s acceptance of GM crops, especially plant-derived edible vaccines. For injected vaccine development, the most cost-effective method is a suspension culture in a closed environment, according to the regulations of good manufacturing practice. Further safety of plant-derived vaccines can be obtained by following the same regulations established for traditional vaccines. In addition, for oral vaccines produced from GM crops, environmental risk assessment and human risk assessment should be performed. For these reasons, plant-derived oral vaccines cannot be called cost efficient compared with traditional vaccines, and the current concern over the use of GM plants is now affecting research in this field.

## Figures and Tables

**Table 1:** Clinical studies of plant-derived vaccines (data from US National Institutes of Health clinical trial).

Application	Study	Status	Sponsor
**Anthrax (1)**	A Phase I Study of the Safety and Immunogenicity of Plant-Derived Recombinant Protective Antigen (rPA) Anthrax Vaccine in Healthy Adults	Phase I (2015), ongoing (NCT02239172)	Fraunhofer, Center for Molecular Biotechnology
**Influenza, Human (3)**	Safety and Immunogenicity of a Recombinant H5N1 Vaccine in Adults	Unknown (2011), (NCT01250795)	Fraunhofer, Center for Molecular Biotechnology
	Immunogenicity, Safety and Tolerability of a Plant-Derived Seasonal Virus-Like-Particle Quadrivalent Influenza Vaccine in Adults	Phase II (2015), ongoing (NCT02233816)	Medicago
	Immunogenicity, Safety and Tolerability of a Plant-Derived Seasonal VLP Quadrivalent Influenza Vaccine in the Elderly Population	Phase II (2015), ongoing (NCT02236052)	Medicago
**Malaria**	Safety and Immunogenicity of Plant-Derived Pfs25 VLP-FhCMB Malaria Transmission Blocking Vaccine in Healthy Adults	Phase I (2015), ongoing (NCT02013687)	Fraunhofer, Center for Molecular Biotechnology
**Gaucher Disease**	A Phase III Trial to Assess the Safety and Efficacy of Plant Cell Expressed GCD in Patients With Gaucher Disease	Phases III, completed (2012) (NCT00376168)	Protalix
**Ebola Virus**	Putative Investigational Therapeutics in the Treatment of Patients With Known Ebola Infection	Phase I,II, recruiting (2015) (NCT02363322)	Zmapp, National Institute of Allergy and Infectious Diseases (NIAID)
**Fabry disease**	An Extension of a Phase I/II, Open Label, Dose Ranging Study of PRX-102 in Adult Fabry Patients	Phase I,II, enrolling (2015) (NCT01769001)	Protalix
**Human Immunodeficiency Virus**	A Safety Study Of A Single Vaginal Administration of P2G12 Antibody In Healthy Female Subjects	Phase I, completed (NCT01403792)	University of Surrey (European Commission)
**Influenza A Subtype H5N1 Infection**	H5-VLP + GLA-AF Vaccine Trial in Healthy Adult Volunteers	Phase I, completed (NCT01657929)	Infectious Disease Research Institute (IDRI), Medicago
**Vitamin B12 Deficiency**	Can Recombinant Human Intrinsic Factor Be Used for Evaluation of the Vitamin B12 Absorption?	Phase II, completed (NCT00279552)	University of Aarhus
**Healthy**	Safety and Immunogenicity of a Recombinant H5N1 Vaccine in Adults	Phase I, unknown (NCT01250795)	Fraunhofer, Center for Molecular Biotechnology

**Table 2:** Published studies on plant-derived antigens for last decade.

Target Disease	Antigen	Host/Expression System	Binary Vector/Agrobacterium Strain	Reference
Malaria	Multi-domain antigen	*N. benthamiana*/transient	pTRAkc-ERH/GV3101	[59]
	pfGAP50	*N. benthamiana*/transient	pMP90RK/GV3101	[65]
	Pfs25, Pfs230_C0	*N. benthamiana*/transient	pTRAkc-ERH/GV3101	[66]
	Multi-domain antigen (PfCSP, PfTRAP, PfCelTOS)	*N. benthamiana*/transient	pMO90RK/GV3101	[67]
	Pf38	*N. benthamiana*/transient	pTRAkc-ERH/GV3101	[68]
	Pfs25	*N. benthamiana*/transient	pGRD4/GV3101	[69]
	PfCP-2.9	Tomato/transgenic	pPS1/LBA4404	[70]
	PyMSP1_19_	*N. benthamiana*/transient	pICH11599/GV3101	[71]
	Pfs230	*N. benthamiana*/transient	pGRD4/GV3101	[72]
	PyMSP4/5	*N. benthamiana*/transient	pICH11599/GV3101	[73]
HIV	2G12	Rice endosperm/transgenic	pTRA/n.a. *	[74]
	Gp120 and gp41	*N. tabacum*/transplastomic	n.a.	[75]
	p24	*A. thaliana*/transgenic *D. carota*/transgenic	pGreen0229/EHA105	[76]
	Multi-HIV (gp120 and gp41)	*N. tabacum*/transplastomic	pKCZ-derived/n.a.	[77]
	MPR of the gp41	*N. benthamiana*/transgenic	pTM096/LBA4404	[78]
	Nef	*N. tabacum*/transgenic	pGreenII0179/GV3101	[79]
	p17/p24	*N. benthamiana*/transient	pTRAc/GV3101	[80]
Influenza	M2e (extracellular domain of matriz protein 2)	*N. benthamiana*/transient	pA7248AMV/GV3101	[81]
	M2e (ectodomain of matrix protein 2)	Duckweed/transgenic	pBI121/n.a.	[82]
	H5 HA	Arabidopsis/transformation	326-Bip/n.a.	[83]
	H5 HA (H5 Hemagglutinin)	*N. benthamiana*/transient	n.a./GV3101	[84]
	H1 & H5 HA	*N. benthamiana*/transient	n.a.	[85]
	H7 HA	*N. benthamiana*/transient	n.a.	[86]
	H1 & H5 HA	*N. benthamiana*/transient	n.a.	[87]
	HA	*N. benthamiana*/transient	n.a.	[88]
	HA	*N. benthamiana*/transient	pGRD4/GV3101	[89]
	HAC1	*N. benthamiana*/transient	n.a.	[90]
	H5 HA	*N. benthamiana*/transient	pTRA/GV3101	[91]
	H1 HA	*N. benthamiana*/-	n.a.	[92]
	M2eHBc	*N. benthamiana*/transient	pGEM/GV3101	[93]
	H5 HA	*N. benthamiana*/transient	n.a.	[94]
	H3 HA	*N. benthamiana*/transient	pBID4/GV3101	[95]
	H3 HA	*N. benthamiana*/transient	pBID4/GV3101	[96]
Avian influenza	rHA0	*N. benthamiana*/transient	magnICON, TMV	[97]
	Hemagglutinin M2	*N. benthamiana*/transient	CMV	[98]
Foot-and-mouth disease	VP1	*N. benthamiana* cell/stable	pBVP1/GV3850	[99]
	VP1	Soybean/transient	pSYCMV-FMDV	[100]
	FMDV VP1	Crotalaria/transgenic	n.a.	[101]
	VP1	Rice/transgenic	n.a.	[102]
	P1-2A3C	Tomato/transgenic	pBin438/GV3101	[103]
	FMDV VP1	*Chenopodium quinoa* *(C. quinoa*)/*N. benthamiana*/transient	CMV-based pBVP1	[104]
Colorectal cancer	GA733-Fc	*N. tabacum*/transgenic	T-easy/LBA4404	[105]
	GA733-FcK	*N. tabacum*/transgenic	pBIN-Plus/LBA4404	[106]
	GA733K, GA733-FcK, and GA733-Fc	*N. tabacum*/transgenic	pBIN-Plus/LBA4404	[107]
	GA733	*N. tabacum*/transgenic *Beta vulgaris*/transient	pBIN-Plus/LBA4404 pICH11599/GV3101	[108]
Mosaic diseases	ALSV and BYMV	*N. benthamiana*/-	n.a.	[109]
Anthrax	PA (Protective antigen)	Tobacco/transgenic	pCHV-RKB/n.a.	[110]
	PA (dIV) (Protective antigen Domain IV)	n.a.	pCambia1303/GV2260	[111]
Polysaccharide encapsulated bacteria	cps3S	*N. tabacum*/transgenic	pCambia2301/GV3101	[112]
Atherosclerosis	ApoB100, CETP	*N. tabacum*/transgenic	n.a.	[113]
West Nile Virus (WNV)	Domain III (DIII) of WNV E protein	*N. benthamiana*/transient	pIC11599 of the MagnICON system/GV3101	[114]
Hemophilia	Factor VIII (FVIII)	*N. tabacum*/transgenic	pLD-CTB/n.a.	[115]
Swine edema	Shiga toxin 2e B (Stx2e)	*L. sativa*/transgenic	pBI121/n.a.	[116]
	Shiga toxin 2e B	*L. sativa*/transgenic, transient *N. tabacum*/transgenic, transient	pBI121/n.a.	[117]
Hemorrhagic colitis & hemolytic-uremic syndrome (*Escherichia coli* O157:H7)	EIT	*N. tabacum*/transgenic	pVSR326/n.a.	[118]
Cholera	CTB	*O. sativa*/transgenic	pZ2028/n.a.	[119]
	CTB-As16	*O. sativa*/transgenic	pGTV/EHA105	[120]
	CTB-FimA	*S. tuberosum*/transgenic	pPCV701/n.a.	[121]
Infectious Bursal Disease	VP2	*N. benthamiana*/transient	pBINPLUS/n.a.	[122]
Bovine rotavirus infection	VP8	*N. tabacum*/transgenic	pBSW-utr/n.a.	[123]
RHD	VP60	*S. tuberosum*/transgenic	pGK/LBA4404	[124]
Diarrhea	Rotavirus VP6	*C. amaranticolor*/transgenic	pGEM/n.a.	[125]
Bovine viral diarrhea	E2	*M. sativa*/transgenic	pBI121/LBA4404	[126]
	(tE2) truncated version of the glycoprotein E2	*N. tabacum*/transient	pK7WG2/EHA105	[127]
Heat labile toxin	B subunit of the heat labile toxin (LTB)	*N. benthamiana*/transient *P. parodii*/transgenic	pBinPlus/n.a.	[128]
	LTB	*N. tabacum*/transgenic *P. Parodii*/transgenic *S. lycopersicum*/transgenic	pBinPlus/LBA9402	[129]
	LTB	*N. benthamiana*/transient *P. parodii*/transgenic *S. lycopersicum*/transgenic	pICH11599/GV3101 pBinPlus/LBA9402 pCLTB/EHA105	[130]
Coccidiosis	EtMIC1 and EtMIC2	*N. tabacum*/transient	pTRA ERH/GV3101	[131]
PRRS	PRRSV matrix (M) protein	Maize callus/transgenic	pAHC25/n.a.	[132]
HPV	HPV16	*N. benthamiana*/transient	pBID4/GV3101	[133]
	HPV16 L1 (fused with LTB)	*N. tabacum*/transgenic	pPNG1014_MCS120/n.a.	[134]
	HPV16 E7	*N. tabacum*/transgenic *N. benthamiana*/transient	pBID4/A4	[135]
Gastroenteritis	VP4 and VP7	*N. benthamiana*/transient	n.a.	[136]
	*OmpA* of *Salmonella typhimurium*	Alfalfa/transgenic	pBI121/LBA4404	[137]
	VP7	*S. tuberosum*/transgenic	pBI121/LBA4404	[45]
Peptic ulceration and gastric cancer (*Helicobacter pylori*)	*Helicobacter pylori* TonB	*A. thaliana*/transgenic	pPCV742/n.a.	[138]
Plague	F1 and V antigens from *Y. pestis*	Lettuce/transgenic	pBI121/LBA4404	[139]
	F1-V	*S. lycopersicum*/transgenic	n.a.	[140]
	F1-V	*N. benthamiana*/transgenic	n.a.	[141]
	F1-V	*N. tabacum*/transgenic	pLD/n.a.	[142]
	F1 and LcrV	*N. benthamiana*/transient	pBID4/A4	[143]
	F1-V	*N. benthamiana*/transient *L. esculentum*/transgenic	pGPTV/LBA4404	[144]
	F1-V	*N. benthamiana*/transient	pICH11599/n.a.	[43]
Tuberculosis	Ag85B/ESAT-6	*N. tabacum*/transgenic	pGEM(T)-easy/C58C1	[145]
	ESAT6:Ag85B	*N. benthamiana*/transient	pBin19/GV3101	[146]
	ESAT-6	*A. thaliana*/transgenic	n.a.	[147]
Newcastle disease	(HN) Hemagglutinin neuraminidase	*N. tabacum*/transgenic	n.a.	[148]
Avian reovirus	σC	*A. thaliana*/transgenic	n.a.	[149]
Diphtheria Tetanus Pertussis (DTP)	DT (diphtheria toxin) TetC (fragment C antigen) PTX S1 (subunit S1 antigen)	*N. tabacum*/transgenic *D. carota*/transgenic	pBinPlus/LBA4404	[150]
RSV (Respiratory Syncytial Virus)	RSV-F	*S. lycopersicum*/transgenic	pJSS-4/n.a.	[151]
Poxvirus infection	pB5	*N. benthamiana*/transient	n.a./GV3101	[152]
SARS	S1	Tobacco/transgenic Lettuce/transgenic	n.a.	[153]
Follicular lymphoma	Idiotypic Ig (tumor-specific antigen)	*N. benthamiana*/transient	magnICON/ICF 320	[154]

* n.a.: Not available; HIV, Human Immunodeficiency; FMDV, Foot-and-mouth disease virus; EIT, Trivalent recombinant protein; CTB, Cholera toxin B subunit; RHD, Rabbit haemorrhagic disease (RHD); HPV, Human papillomavirus; SARS, Severe acute respiratory syndrome.

**Table 3:** Published studies on plant-derived hepatitis B virus (HBV) antigens and expression levels.

Antigen	Host Plant/Expression System	Binary Vector/Agrobacterium Strain	Promoter/Targeting	Maximum Yield	Application and Results	Reference
**S-HBsAg**	*N. tabacum*/transgenic	pBI121/LBA4404	35S/n.a. *	0.01% of TSP	Injection, IgA, IgG and IgM antibodies in serum	[163,175]
	*N. tabacum*/transgenic	pGA482/LBA4404	35S/n.a.	0.05% of TSP	n.a.	[176]
	*N. tabacum*/transgenic	pBI121/EHA105	*UBQ3*/ER	20 ng/g FW	n.a.	[172]
	*N. tabacum*/transgenic	pKHBSBAR/EHA105	35S/n.a.	10 µg/g FW	Injection, IgG antibodies in serum (maximum 765 mIU/mL)	[177,178]
	*N. tabacum*/transgenic	pE1802,pE1945/LBA4404	(Aocs)_3_AmasPmas/n.a.	0.06% of TSP	n.a.	[179]
	*N. tabacum*/transgenic	pAMPAT-MCS/GV3101	D35S/n.a.	2–26 ng/g FW	Oral, IgA, IgG antibodies in serum	[26,180]
	*N. tabacum*/transgenic	pBM/LBA4404	D35S/n.a.	0.01%–0.05% of TSP	n.a.	[181]
	*N. benthamiana*/transient	pICH11599/GV3101	Act2/ER	295 µg/g FW	Injection, IgG antibodies in serum (maximum 830 mIU/mL)	[30]
	*N. benthamiana*/transient	pIBT210/LBA4404	35S/n.a.	0.05% of TSP	Injection, IgA and IgG antibodies in serum (maximum 346 mIU/mL)	[182]
	NT-1 cell/transgenic	pBI121/EHA105	UBQ3/ER	2 µg/g FW	n.a.	[173]
	NT-1 cell/transgenic	pBI121/EHA105	UBQ3/ER	31 µg/L culture medium	n.a.	[183]
	NT-1 cell/transgenic	pHB155/n.a.	D35S/n.a.	8 µg/g FW (2 mg/L culture medium)	n.a.	[174]
	NT-1 cell/transgenic	pGPTV-KAN/LBA4404	35S/ER	0.005%–0.023% of TSP	Injection, IgG antibodies in serum (maximum 4 mIU/mL)	[184]
	Potato/transgenic	pDES20/GV3101	D35S/n.a.	1 µg/g FW (tuber)	n.a.	[164]
	Potato/transgenic	pBI101/LBA4404	35S/n.a.	6 µg/g FW (tuber)	Injection	[185]
	Potato/transgenic	pBI101/LBA4404	PAT/n.a.	1.1 µg/g FW (tuber)	Oral, IgG antibodies in serum (maximum 1680 mIU/mL)	[165]
	Potato/transgenic	pHB114/LBA4404	35S/n.a.	11 µg/g FW (tuber)	Oral, IgG antibodies in serum (maximum 4785 mIU/mL)	[41]
	Potato/transgenic	pBI121/LBA4404	PAT/n.a.	0.09% of TSP (tuber)	Oral, IgG antibodies in serum (maximum 800 mIU/mL)	[167]
	Potato/transgenic	pHB114/LBA4404	35S/n.a.	8.35 µg/g FW (tuber)	Oral, IgG antibodies in serum (maximum 3300 mIU/mL)	[166]
	Potato/transgenic	pBM/LBA4404	D35S/n.a.	0.05% of TSP (tuber)	Oral, IgG antibodies in serum (maximum 350 mIU/mL)	[186]
	Potato/transgenic	pBM/LBA4404	35S/n.a.	1 µg/g FW (tuber)	Oral, IgG antibodies in serum (maximum 350 mIU/mL)	[186]
	Lettuce/transgenic	pROK/LBA4404	35S/n.a.	5.5 ng/g FW	n.a.	[187]
	Lettuce/transgenic	pGPTV-BAR/EHA105	35S/n.a.	60 µg/g FW	Oral, IgA and IgG antibodies in serum (maximum 38 mIU/mL), Oral (PBS-suspended lyophilized tissue), IgG antibodies in serum (maximum 300 mIU/mL)	[188,189]
	*A. thaliana*/transgenic	pAMPAT-MCS/GV3101	D35S/n.a.	3–15 ng/g FW	Oral, IgA, IgG antibodies in serum	[26,180]
	Soybean cell/transgenic	pHB155 vector/n.a.	D35S/n.a.	(74 μg/g FW) 22 mg/L culture medium	n.a.	[174]
	Soybean cell/transgenic	pMSI164/EHA105	UBQ3/ER	700 ng/g FW	n.a.	[190]
	Banana/transgenic	pBI121/EHA105	EFE ER	38 ng/g FW (leaf)	n.a.	[191]
	Lupin/transgenic	pROK/C58	35S/n.a.	150 ng/g FW (Callus)	Oral, IgG antibodies in serum (maximum 19 mIU/mL)	[187]
	Lupin/transgenic	pROK/GV3101, EHA105, LBA4404	35S/n.a.	2.5–6 µg/g FW (Callus)	n.a.	[192]
	Maize/transgenic	n.a./EHA101	3xglb1/Cell wall	0.51% of TSP (seed)	n.a.	[168]
	Maize/transgenic	n.a./EHA101	Glob1/Cell wall	0.46% of TSP (seed)	Oral (germ, bioencapsulated), IgA and IgG antibodies in serum (maximum 4632 mIU/mL)	[193]
	Maize/transgenic	n.a./EHA101	3kbglb1/Cell wall	0.41% of TSP (seed)	Oral (germ, wafer feeding), IgA and IgG antibodies in serum	[194]
	Maize/transgenic	n.a./EHA101	Glob1/Cell wall	0.46% of TSP (seed)	Oral (germ, wafer feeding), IgA and IgG antibodies in serum	[195]
	Cherry tomatillo/transgenic	pCAMBIA1301/EHA105	35S/n.a.	10 ng/g FW (fruit)	Oral, IgG antibodies in serum	[196]
	Tomato/transient	pBI121/EHA105	EFE/ER	0.5 µg/g DW (leaf)	n.a.	[169]
	Tomato/transgenic	pBINPLUS-ARS/LBA4404	35S/ER	n.a.	n.a.	[191]
	Tomato/transgenic	pBM/LBA4404	D35S/n.a.	0.01%–0.05% of TSP (leaf)	n.a.	[181]
	Tomato/transgenic	pCAMBIA1301/EHA105	35S/n.a.	n.a.	n.a.	[197]
	Tomato/transgenic	pBINPLUS-ARS/LBA4404	35S/n.a.	0.3 µg/g DW (fruit)	Oral, IgA and IgG antibodies in serum (maximum 300 mIU/mL)	[193]
	Carrot cell/transgenic	pPCV812/n.a.	MAS/n.a.	25 ng/g FW	n.a.	[198]
	*Laminaria japonica*/transgenic	pCAT/n.a.	SV40/n.a.	0.05%–0.25% of TSP	n.a.	[199]
	Peanut/transgenic	pCAMBIA1301/EHA105	35S/n.a.	240 ng/g FW (bud)	Injection	[200]
**M-HBsAg**	*N. tabacum*/transgenic	n.a.	n.a.	10 µg/g FW	Injection, IgG antibodies in serum (maximum 1165 mIU/mL)	[177]
	Tobacco/transgenic	pGPTV-BAR/EHA105	35S/n.a.	12–21 µg/g FW	n.a.	[201]
	*N. benthamiana*/transient	pIBT210/LBA4404	35S/n.a.	0.04% of TSP	Injection, IgA and IgG antibodies in serum (maximum 1165 mIU/mL)	[182]
	Potato/transgenic	pBI121/LBA4404	PAT/n.a.	0.012% of TSP (tuber)	Oral, IgG antibodies in serum (maximum 800 mIU/mL), Oral, IgA and IgG antibodies in serum (maximum 558 mIU/mL)	[167,202]
	Lettuce/transgenic	pGPTV-BAR/EHA105	35S/n.a.	2–23 µg/g FW	n.a.	[201]
	Tomato/transgenic	pBINPLUS-ARS/LBA4404	35S/n.a.	0.002% of TSP (fruit)	n.a.	[203]
	Tomato/transgenic	pBINPLUS-ARS/AGLO	35S/n.a.	0.003%–0.021% of TSP (fruit)	Oral (freeze-dried material), IgG antibodies in serum	[204,205]
	Carrot/transgenic	pBINPLUS-ARS/n.a.	35S/ER	42 ng/g FW (leaf)	n.a.	[206]
**L-HBsAg**	Tobacco/transgenic	pGPTV-BAR/EHA105	35S/n.a.	4–13 µg/g FW	n.a.	[201]
	Lettuce/transgenic	pGPTV-BAR/EHA105	35S/n.a.	3–20 µg/g FW	n.a.	[201]
	Tomato/transgenic	pYPX143/LBA4404	2A11/n.a.	0.5 µg/g FW (Fruit)	n.a.	[171]
**Hepatitis B core antigen**	*N. tabacum*/transgenic	pBIN19/LBA4404	E12Ω/n.a.	24 µg/g FW	n.a.	[207]
	*N. tabacum*/transient	pICH11599/GV3101	35S/n.a.	2.38 mg/g FW	Injection and Oral, IgA and IgG antibodies in serum	[208]
	*N. benthamiana*/transient	pBINPLUS/LBA4404	35S/n.a.	1 mg/g FW	n.a.	[209]
	*N. benthamiana*/transient	pBY023/LBA4404	35S/n.a.	0.18 mg/g FW	n.a.	[210]
	*N. benthamiana*/transient	pEAQ-HT/LBA4404	35S/n.a.	0.2–1 mg/g FW	n.a.	[211]

* n.a.: Not available; TSP, total soluble protein; UBQ3, ubiquitin 3; ER, endoplasmic reticulum; FW, fresh weight; PAT, patatin; PBS, phosphate buffered saline; EFE, ethylene-forming enzyme; DW, dry weight.
